# Longer Interscan Times in OCT Angiography Detect Slower Capillary Flow in Diabetic Retinopathy

**DOI:** 10.1016/j.xops.2022.100181

**Published:** 2022-06-13

**Authors:** Yoshihiro Kaizu, Shintaro Nakao, Tomomi Soda, Juun Horie, Iori Wada, Muneo Yamaguchi, Atsunobu Takeda, Koh-Hei Sonoda

**Affiliations:** 1 Department of Ophthalmology, Graduate School of Medical Sciences, Kyushu University, Fukuoka, Japan; 2 Department of Ophthalmology, National Hospital Organization, Kyushu Medical Center, Fukuoka, Japan; 3 Clinical Research Institute, National Hospital Organization, Kyushu Medical Center, Fukuoka, Japan; 4 Canon Inc., Tokyo, Japan

**Keywords:** Capillary dropout, Diabetic macular edema, Microaneurysms, VISTA, AOSLO, adaptive optics scanning laser ophthalmoscopy, DCP, deep capillary plexuses, DME, diabetic macular edema, DR, diabetic retinopathy, FA, fluorescein angiography, FAZ, foveal avascular zone, GA, geographic atrophy, IPL, inner plexiform layer, NDR, no DR, NPDR, nonproliferative DR, OCTA, optical coherence tomography angiography, PDR, proliferative DR, SCP, superficial capillary plexuses, SD-OCT, spectral domain optical coherence tomography, VISTA, variable interscan time

## Abstract

**Purpose:**

To investigate the detection of slower retinal capillary blood flow using commercial OCT angiography (OCTA) with a longer interscan time in diabetic retinopathy (DR).

**Design:**

Observational, prospective, cross-sectional study.

**Participants:**

A total of 62 eyes from 39 subjects with diabetes mellitus and 10 eyes from 9 healthy subjects.

**Methods:**

Commercial spectral domain-OCT was used to obtain 3 × 3-mm fovea-centered OCTA images of all eyes with 3 different interscan times (4.3, 5.7, and 8.6 ms). For each interscan time, OCTA imaging was performed 5 consecutive times, and a ×5 averaged image was obtained. Capillary flow density and visualization of retinal capillaries in the superficial and deep capillary plexuses (SCPs and DCPs, respectively) were compared between the 3 averaged images from the 3 different interscan times.

**Main Outcome Measures:**

Capillary flow density and visualization of foveal capillaries in 3 images with different interscan times.

**Results:**

Forty-five eyes of 34 patients were analyzed. There was no significant difference in the flow density of the SCP and DCP between the 3 images with different interscan times in all the DR stages. Some capillaries including microaneurysms that could not be observed at 4.3 ms could be observed at 5.7 or 8.6 ms. There were significantly more capillaries with difference points between the 3 images in the group with DR than in the group without DR (*P* < 0.01). The morphology of some microaneurysms also changed with longer interscan times.

**Conclusions:**

OCTA with longer interscan times revealed slower flow points in capillaries and more accurate visualization and morphology of microaneurysms in DR.

Diabetic retinopathy (DR) remains the leading cause of blindness and severe vision loss despite recent advances in diagnosis and therapies.^1^ It is characterized by neurological and vascular damage due to hyperglycemia and abnormal blood sugar fluctuations.^2^^,^^3^ Among its pathogenesis, vascular abnormalities at the capillary level, such as microaneurysms and capillary dropouts, are crucial for the progression of DR. Therefore, accurate vascular imaging is important for understanding the pathogenesis of DR and determining its treatment strategy. Various studies using fluorescein angiography (FA) and other devices have shown that abnormalities in blood flow, besides vascular abnormalities, occur during DR.^4^^,^^5^

OCT angiography (OCTA) visualizes retinal microvasculature at the capillary level by recognizing the dynamic change of erythrocytes.^6^^,^^7^ Various studies have reported the clinical usefulness of OCTA in DR by detecting and quantifying retinal vascular abnormalities (e.g., microaneurysms, neovascularization, and nonperfused areas).^8^, ^9^, ^10^, ^11^, ^12^, ^13^, ^14^, ^15^, ^16^ However, conventional OCTA can only detect blood flow with a velocity dependent on the interscan time, indicating that vascular abnormalities with slower blood flow are undetectable.^7^

Choi et al developed a technology called variable interscan time (VISTA), based on the concept originally proposed by Tokayer et al,^17^ to delineate relative blood flow velocity. This technique was first introduced in cases of age-related macular degeneration with geographic atrophy (GA).^17^^,^^18^ They created and compared 2 images using 2 different interscan times (1.5 and 3.0 ms) and found that some choroidal vessels, particularly around the margin of the GA, that could not be visualized with a shorter interscan time could be visualized with a longer interscan time image. Furthermore, Rebhun et al^19^ showed possible heterogeneous blood flow speed in the polyps of polypoidal choroidal vasculopathy. Recently, Arya et al^20^ examined blood flow in 13 patients with DR using VISTA and reported the alteration of blood flow in DR-associated microvasculopathy. Ploner et al^21^ also reported a method of using color mapping for VISTA algorithm to display relative retinal flow velocity.

Spaide et al^22^ previously reported that repeated OCTA scans show the different images of microaneurysms in eyes with DR. Unfortunately, the current commercial OCTA system cannot detect all microaneurysms detected by FA.^8^^,^^23^, ^24^, ^25^ We reported that the averaging process of multiple OCTA images stabilizes the visualization of microaneurysms and improves detection in DR,^26^ suggesting that OCTA averaging is specifically effective for the visualization of microaneurysms with intermittent blood flow. Moreover, we observed that some microaneurysms were not visualized even in averaged OCTA images, leading to the hypothesis that these microaneurysms may have slow blood flow that is below the blood flow detection threshold of the OCTA system.^26^ In this study, we performed OCTA imaging using a longer interscan time setting in a commercial OCTA device and investigated the differences in the visibility of retinal capillaries in the eyes of healthy subjects and patients with diabetes with various DR stages.

## Methods

### Participants

This study was approved by the institutional ethics committees of Kyushu University Hospital (28-473, UMIN 000028656) and Kyushu Medical Center (20A174) and was performed in accordance with the ethical standards established by the Declaration of Helsinki. Written informed consent was obtained from all participants in this study.

### Patient Population

We prospectively enrolled 62 eyes of 39 patients with diabetes with varying severities of DR and 10 eyes of 9 nondiabetic subjects. The inclusion criterion for visual acuity was 20/32 or higher. All participants underwent the following general ophthalmologic examinations at Kyushu University Hospital or Kyushu Medical Center between October 2018 and October 2021: measurement of best-corrected visual acuity, slit-lamp microscopy, color fundus photography, and OCT imaging. We excluded eyes with any other ocular disease that may lead to microvascular disturbance in the retina or choroid (e.g., age-related macular degeneration, retinal vascular occlusion, glaucoma) and severe diabetic macular edema (DME) that significantly affects visual acuity.

### OCT Angiography

All participants underwent OCTA examination using spectral-domain (SD)-OCT. This device has an A-scan rate of 70 000 scans per second and a wavelength of 855 nm. The vertical and axial resolutions were 20 μm and 3 μm, respectively. The segmentation lines were defined as follows: (a) for the superficial capillary plexus (SCP), the inner and outer boundaries were set at the inner limiting membrane and 50 μm below the inner plexiform layer (IPL), respectively, while (b) for the deep capillary plexus (DCP), the inner and outer boundaries were set at 50 μm below the IPL and outer plexiform layer, respectively. The scanning area was set at a 3 × 3-mm fovea-centered region. We applied 3 interscan time settings (4.3, 5.7, and 8.6 ms). OCT angiography imaging was performed 5 consecutive times for each IT setting (Table 1). Low-quality images with a signal strength of < 5 or image artifacts were excluded from the present study. For each interscan time setting, 5 high-quality single-OCTA images that satisfied the aforementioned acceptance criterion were obtained (i.e., a total of 15 OCTA images were obtained for each eye). Finally, using the 5 best single-OCTA images, averaged OCTA images were generated by an image-averaging software program installed on the OCT-A1, as previously described.^26^ In this study, SCP and DCP images were used for analysis.

**Table 1:** Flow Density in Whole 3 × 3-mm Area of Each Interscan Time Setting (One-Way Analysis of Variance)

		Interscan Time (millisecond)			*P* Value
		4.3	5.7	8.6	
Healthy (n = 9)	SCP	0.413 ± 0.012	0.415 ± 0.010	0.416 ± 0.010	0.89
	DCP	0.420 ± 0.006	0.422 ± 0.007	0.422 ± 0.006	0.64
NDR (n = 13)	SCP	0.403 ± 0.007	0.406 ± 0.007	0.408 ± 0.006	0.15
	DCP	0.417 ± 0.005	0.419 ± 0.006	0.421 ± 0.008	0.30
NPDR (n = 11)	SCP	0.409 ± 0.006	0.409 ± 0.008	0.407 ± 0.010	0.81
	DCP	0.413 ± 0.012	0.415 ± 0.009	0.413 ± 0.012	0.86
PDR (n = 12)	SCP	0.375 ± 0.037	0.376 ± 0.035	0.378 ± 0.034	0.98
	DCP	0.381 ± 0.037	0.388 ± 0.031	0.396 ± 0.027	0.55

DCP = deep capillary plexus; NDR = no diabetic retinopathy; NPDR = nonproliferative diabetic retinopathy; PDR = proliferative diabetic retinopathy; SCP = superficial capillary plexus.

### Image Evaluation and Analysis

Three types of averaged OCTA images (interscan time = 4.3, 5.7, and 8.6 ms, respectively) were cropped by a 1.5-mm-diameter circle centered on the images. The outline of the foveal avascular zone (FAZ) was manually contoured and measured using ImageJ, version 1.51f. We set the donut-shaped area (i.e., a 1.5-mm-diameter circle centered on the images without the FAZ) as the evaluation area. Each OCTA image (SCP, DCP, and interscan time = 4.3, 5.7, and 8.6 ms) was converted to binarized images, and the flow density of each evaluation area was calculated using ImageJ. Two independent retinal specialists (Y.K. and S.N.) evaluated the differences in the imaged foveal capillaries among the 3 OCTA images. When the evaluations of the 2 observers differed, a third observer (I.W.) evaluated the capillary visualization. The k coefficient was 0.73 (95% confidence interval, 0.37–1.0; *P* < 0.0001) and 0.87 (95% confidence interval, 0.70–1.0; *P* < 0.0001) for the evaluation of SCP and DCP, respectively. The number of different points per flow density in the evaluation area was calculated.

### Microaneurysms Evaluation

In DR cases, all microaneurysms within the analysis area were classified according to a previously reported morphological classification.^27^^,^^28^ The morphology of the microaneurysms was subsequently compared between the 3 images with different interscan times.

### Statistical Analysis

All data are expressed as mean (SD). All statistical analyses were performed using JMP, version 15.0. Non-normally distributed data were analyzed using nonparametric statistics. Statistical significance was set at *P* < 0.05.

## Results

Out of the 62 eyes with DR, 26 (41.9%) were excluded because of low image quality due to cataract, vitreous hemorrhage, poor fixation, or image artifacts. One eye (10.0%) was also excluded from the nondiabetic group. Consequently, 45 eyes of 34 patients could be analyzed (mean age, 55.2 ± 12.8 years; 21 male and 13 female; 9 eyes without diabetes, 13 eyes with no DR [NDR], 11 eyes with nonproliferative DR [NPDR], 12 eyes with proliferative DR [PDR]).

### Flow Density of OCTA Imaging with Different Interscan Times

**Figure 1:**
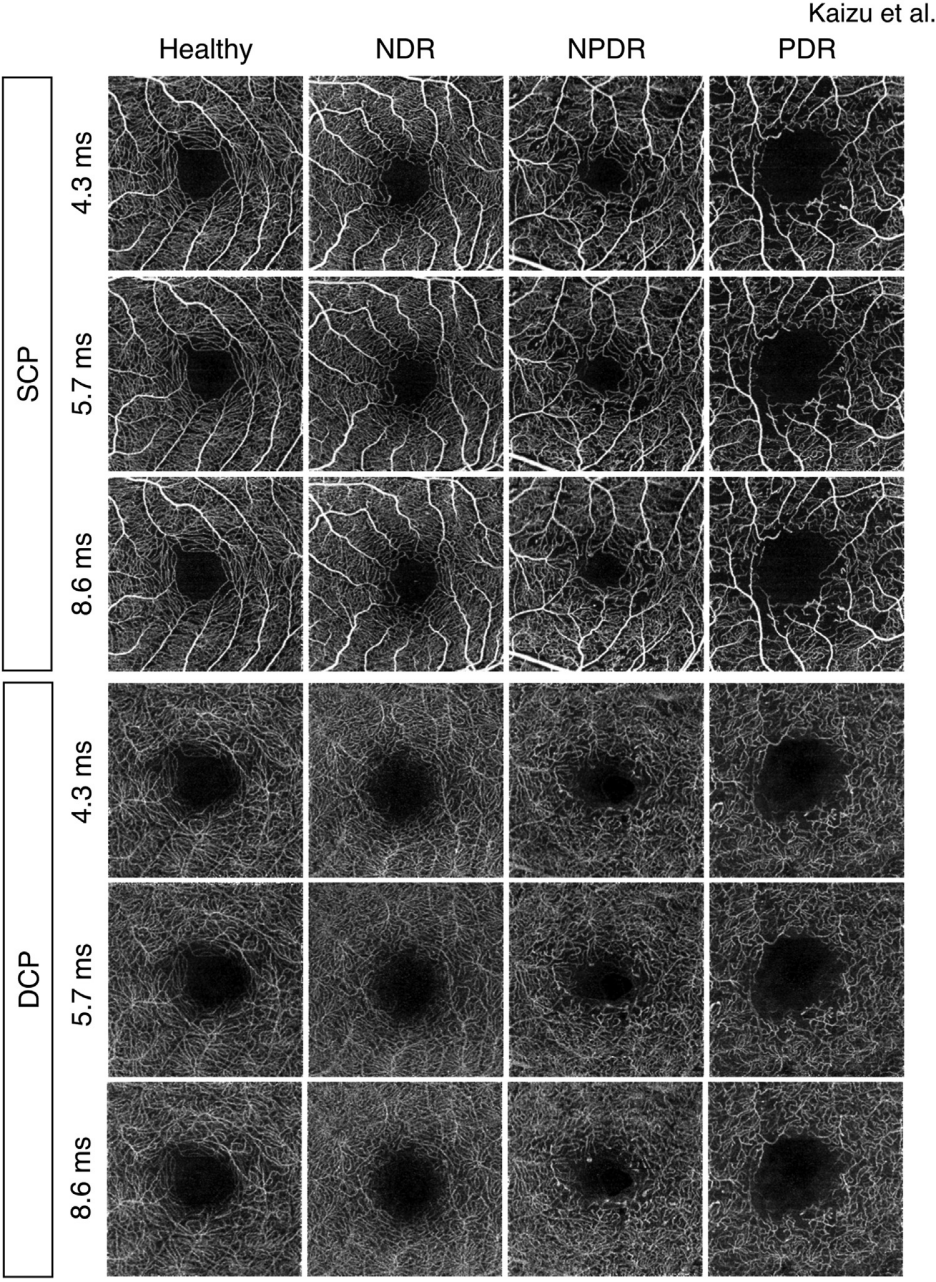
Representative OCT angiography (OCTA) images (3 × 3 mm) with 3 different interscan times (4.3, 5.7, and 8.6 ms) obtained from a healthy participant (76-year-old female, left eye), patient with no diabetic retinopathy (NDR) (62-year-old female, left eye), patient with nonproliferative diabetic retinopathy (NPDR) (66-year-old male, left eye), and patient with proliferative diabetic retinopathy (PDR) (44-year-old male, right eye). Averaged OCTA images were generated by image-averaging software installed on the OCT-A1 using the 5 best single-OCTA images. DCP = deep capillary plexuses; SCP = superficial capillary plexuses.

We first examined the flow density of OCTA-averaged images at 3 different interscan times separately for the SCP and DCP. Retinal flow density in the SCP and DCP was not significantly different among the 3 images at all stages (Fig 1).

### Differences in OCTA Imaging with Different Interscan Times

**Figure 2:**
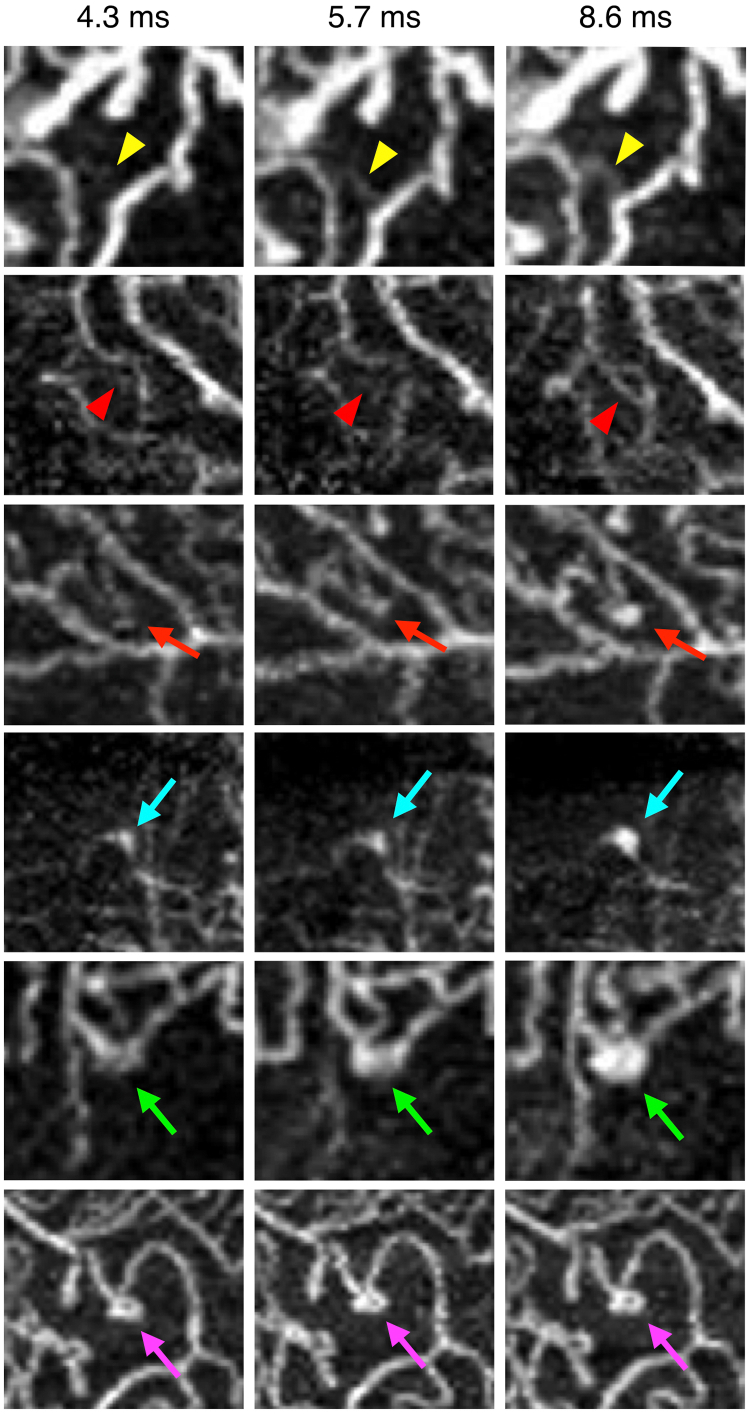
Comparison of OCT angiography (OCTA) images with 3 different interscan times (4.3, 5.7, and 8.6 ms). Yellow arrowheads indicate a retinal capillary that cannot be observed at 4.3 ms but can observed at 5.7 and 8.6 ms. Red arrowheads indicate a retinal capillary that cannot be observed at 4.3 and 5.7 ms but can be observed at 8.6 ms. Red arrows indicate a microaneurysm that cannot be observed at 4.3 and 5.7 ms but can be observed at 8.6 ms. Blue and green arrows indicate a microaneurysm that can be classified as a focal bulge and fusiform type at 4.3 and 5.7 ms, respectively, and can be classified as a saccular type at 8.6 ms. Pink arrows indicate a saccular type–microaneurysm that does not change its morphology at 3 different interscan times.

**Figure 3:**
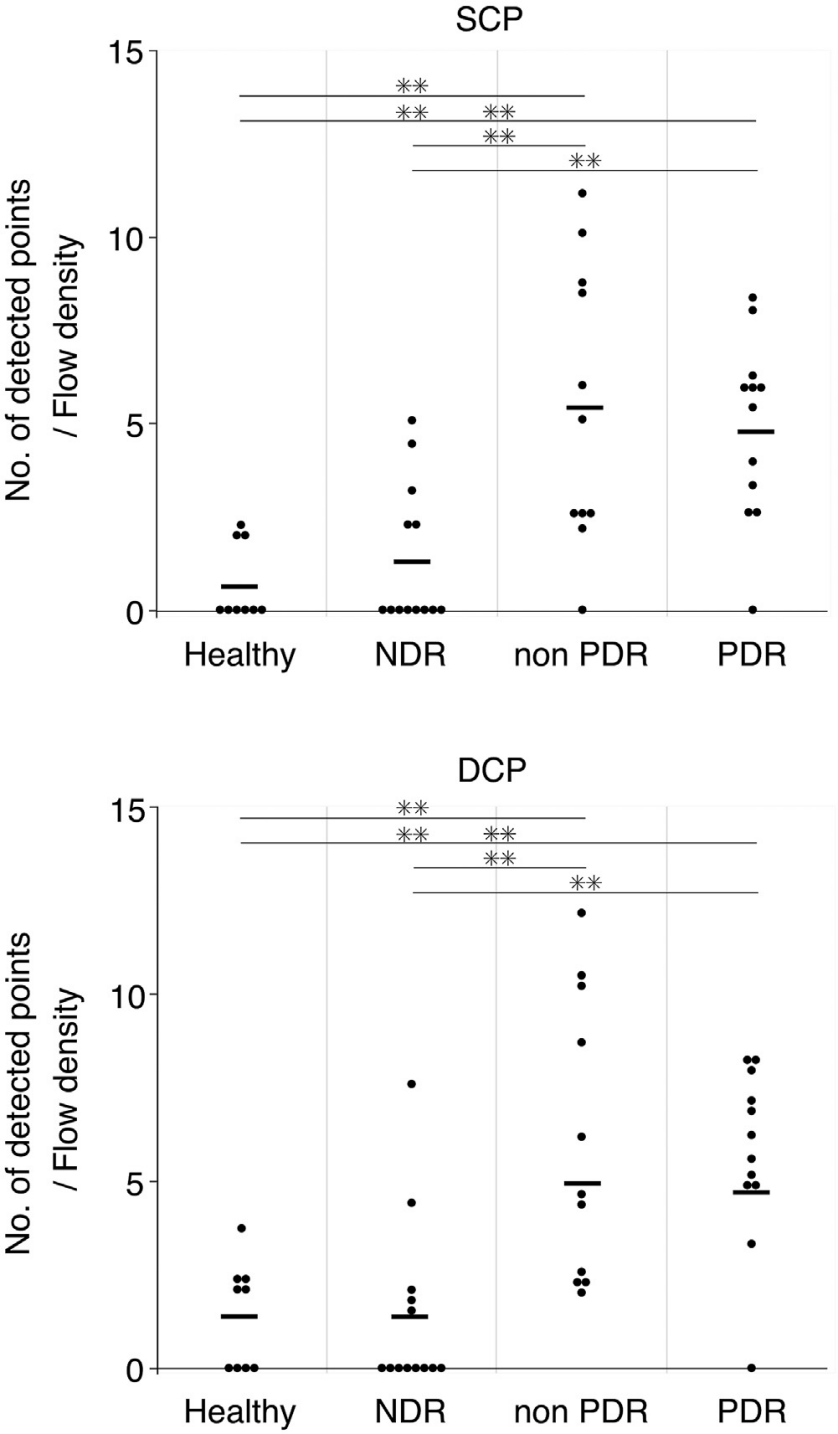
Comparison of the capillary visualization among 3 images with 3 different interscan times (4.3, 5.7, and 8.6 ms). Figures show the different detected points per retinal capillary flow density of the superficial and deep capillary plexus (SCP and DCP, respectively) among the 4 groups (healthy, no diabetic retinopathy [NDR], nonproliferative diabetic retinopathy [NPDR], and proliferative diabetic retinopathy [PDR] subjects; n = 9, 13, 11, and 12, respectively). ∗∗*P* < 0.01, Tukey–Kramer test.

Afterward, we compared the capillary visualization among the 3 images at different interscan times. There were some capillaries that were not imaged with the 4.3-ms interscan time but were imaged with the 5.7- and 8.6-ms interscan times. There were also capillaries that were not observed with the 4.3- and 5.7-ms interscan time images but could be observed with the 8.6-ms interscan time image (Fig 2). The different imaging points in both the SCP and DCP were significantly higher in the NPDR and PDR groups than in the healthy and NDR groups (*P* < 0.01, Fig 3).

### Microaneurysms Visualization with Different Interscan Times in OCTA Imaging

We observed some microaneurysms that were not imaged with shorter interscan times but with longer interscan times (Fig 2). In addition, some microaneurysms had different morphologies depending on the interscan time (Fig 2). However, some microaneurysms, whose morphology did not change at any of the interscan times, could also be observed (Fig 2).

## Discussion

In this study, we prospectively investigated the differences in capillary visualization in patients with DR using 3 interscan times that were longer than those previously reported using VISTA.^18^, ^19^, ^20^, ^21^ We performed image averaging to eliminate the differences in vascular flow in each imaging and found undetectable capillaries at the 4.3-ms interscan time but not at the 5.7-ms or 8.6-ms interscan time in healthy subjects and in patients with diabetes. Slower flow was more frequently detected in DR eyes than in NDR and nondiabetic eyes in both the SCP and DCP. In some cases of DR, microaneurysms were undetected using a shorter interscan time; however, they were visualized using a longer interscan time. In addition, some cases of microaneurysms showed different morphologies at different interscan times. These results indicate that in DR, there is slower blood flow that cannot be detected by OCTA in a conventional setting. Therefore, prolonging the interscan time enables accurate detection and visualization of capillaries with slower blood flow.

It has been reported that enlargement of the FAZ correlates with decreased visual acuity, suggesting that blood flow around the FAZ is important for visual function.^29^ In this study, we investigated the foveal capillaries around the FAZ and detected areas of slower blood flow in the fovea of patients with DR. Arya et al^20^ also reported a decrease in blood flow around the FAZ as the disease progressed, which is consistent with our observation. However, the clinical significance of capillaries with slower blood flow is currently unclear. A previous study using adaptive optics scanning laser ophthalmoscopy (AOSLO) observed leukocyte-induced erythrocyte aggregation in parafoveal capillaries in healthy eyes.^30^ In addition, various studies using DR animal models suggest that leukocyte adhesion via adhesion molecules, such as intercellular adhesion molecule-1, is involved in capillary occlusion during DR progression.^31^^,^^32^ This suggests that such hypoperfusion in DR may lead to capillary dropout and FAZ enlargement in the future. However, longitudinal studies are required to confirm this hypothesis.

Surprisingly, in the present study, no significant differences in retinal flow density were observed between the 3 interscan times for both SCP and DCP. This might indicate that the current commercial setting is sufficient in quantifying retinal vascular density. However, while retinal vessels are three-dimensional, OCTA flow density is a two-dimensional measurement; therefore, significant differences may be able to be observed using volumetric analysis.^12^

The VISTA technique of Arya et al^20^ used different interscan times (1.5 and 3.0 ms) in a single imaging session to examine retinal blood flow. This interscan time might be shorter than the preset interscan time in commercial OCTA devices. In contrast, we used longer interscan times to study eyes with DR to detect capillaries with slower flow that were undetectable with a commercial OCTA. In our previous study on healthy eyes, we observed capillaries that were visualized by AOSLO but not by OCTA,^33^ indicating the existence of undetectable slower capillaries in a commercial OCTA setting. However, the slower blood flow detected in normal eyes may be due to other factors such as aging. Therefore, we cannot conclude that all of the slow capillaries detected in this study are pathological phenomena.

Previous reports have suggested a significant decrease in blood flow in eyes with DR despite observations concerning larger retinal vessels.^4^^,^^5^ Furthermore, in the present study, the number of capillaries with decreased blood flow detected with a longer interscan time was significantly higher in eyes with DR than in those without. This indicates that retinal blood flow is slower in eyes with DR even at the capillary level than in those without. In this study, we also found that there was no difference between NPDR and PDR at the capillary level, which is the main pathological locus of DR. This may indicate that some cases of PDR may be the result of an already completed vascular occlusion. However, further studies with a larger number of patients are needed.

Microaneurysms are one of the major causes of DME^34^; however, it is known that DR includes various morphological types of microaneurysms, according to pathological and AOSLO studies.^27^^,^^35^ However, detailed morphologies were unobservable in FA due to fluorescence leakage. In contrast, it has been reported that microaneurysms can be morphologically classified by OCTA.^26^^,^^28^ However, Spaide et al^22^ reported that the morphology varies with each OCTA imaging session. We also reported some microaneurysms that could not be imaged by OCTA even with image averaging, suggesting that these could be because of their slower blood flow.^26^ In this study, we observed that there are microaneurysms that cannot be imaged only by image averaging but can be imaged with longer interscan times. Arya et al^20^ also reported that 57.3% of microaneurysms have relatively slow blood flow. Furthermore, in this study, we observed that the morphology of some microaneurysms was different with longer and shorter interscan time imaging. This suggests that blood flow velocity is nonconstant in microaneurysms, which may be consistent with the presence of turbulent microaneurysms in our previous AOSLO observation.^23^ In future clinical practice, blood flow should be considered in determining the types of microaneurysms that are clinically significant.

This study has several limitations. First, the sample size was small. Second, patients with poor visual acuity were excluded, even though some patients with DR have severe vision loss. Third, the examined field of view was small in the fovea, although vascular abnormalities in DR can be observed anywhere in the retina. Fourth, patients with previous treatment for DME (e.g., anti-vascular endothelial growth factor therapy or vitrectomy despite no recent treatment) were included despite the fact that these therapies might affect vascular flow. Fifth, the current method used requires > 15 images to be taken, which takes a long time and limits the number of images that can be analyzed. Therefore, new technologies for clinical use must be improved to reduce the total imaging time. Future studies are needed to investigate whether these treatments can affect the improvement of the detection of slower blood flow using OCTA with longer interscan times.
